# The Source of the River as a Nursery for Microbial Diversity

**DOI:** 10.1371/journal.pone.0120608

**Published:** 2015-03-24

**Authors:** Luiz Felipe Valter de Oliveira, Rogério Margis

**Affiliations:** 1 Programa de Pós-graduação em Genética e Biologia Molecular, Universidade Federal do Rio Grande do Sul, UFRGS, Porto Alegre, Brazil; 2 Centro de Biotecnologia and Departamento de Biofísica, Universidade Federal do Rio Grande do Sul, UFRGS, Porto Alegre, Brazil; U. S. Salinity Lab, UNITED STATES

## Abstract

Bacteria are highly diverse and ubiquitous organisms that play a key role as drivers for ecosystem processes. The application of NGS (next-generation sequencing technologies) for 16S analysis has been broadly used for understanding bacterioplankton composition and structure. Most of studies conducted on aquatic ecosystems with 16S NGS have been in seawater and lakes. A few studies using NGS have been conducted in river environments and have suggested the presence of a bacterial seed-bank. We performed 16S highly variable V4 region high-throughput analysis in the Sinos River, which is located in one of most important Brazilian industrial centers. This region has several contrasts in its environmental characteristics, presenting a longitudinal gradient of eutrophication and making it a remarkable study site for observing the dynamics of bacterioplankton. We demonstrated consistent evidence for the existence of a bacterial seed-bank and its longitudinal persistence. Seasonal shifts reinforce the importance of the source of the river in maintaining the bacterial seed-bank that spreads throughout the river. Therefore, the preservation of the source of the river is important not only for hydrologic reasons but also to maintain the microbial composition and the ecological integrity of the river.

## Introduction

The large domain of prokaryotic organisms encompassing the microbiome is highly diverse and ubiquitous, playing key roles as drivers of the major ecosystem processes [1]. In recent years, the application of NGS to 16S analysis has changed the sensitivity and throughput of microbiological analysis and has been widely used for understanding bacterioplankton [2,3]. For seawater, the most studied aquatic ecosystem, some recent studies have described several patterns of bacterial diversity [4–6]. Seasonal and environmental changes can affect bacterioplankton community structures [7]. However, there are microbial seed-banks that persist throughout seasons [8] and are spread throughout the global ocean [9]. Studies from lakes and streams represent the majority of knowledge of freshwater bacterioplankton. These studies have been important for understanding the structure, composition and dynamics of microbial diversity [10–13] as well as microbial responses to environmental disturbances [14,15].

A few high-throughput 16S-sequencing studies have also been conducted in river environments. Bacterioplankton in six rivers from circumpolar locations have been shown to shift synchronously, in correlation with seasonal variations [16]. More recently, the bacterioplankton community structure has been shown to shift in the Mississippi River with changes in relative abundance, rather than the presence/absence of OTUs (Operational Taxonomic Units), suggesting the existence of a bacterial seed-bank in the river ecosystem [17].

The Sinos River is located in one of most important Brazilian industrial centers [18] and is divided into three sections: the upper, middle and lower courses, moving downriver. This classification is paralleled by an increased gradient of pollution and human population density. The upper course is approximately 25 km long and flows very rapidly; it has lower human population density with low/moderate impacts from domestic sewage and agricultural waste. The middle course, approximately 125 km long with moderate water flow, is more environmentally impacted, with large areas of rice crops and cattle farms and increased human population density. The lower course, which stretches over 50 km and has very slow water flow, has a greater number of industries and an urban population of more than 600,000 habitants [18–20]. The Sinos River also has a history of catastrophic events caused by domestic and industrial pollution, which resulted in the death of 90 tons of fish in 2006. These contrasts in its environmental characteristics and the presence of a eutrophication gradient along the Sinos River make it a remarkable study site for observing the dynamics of bacterioplankton at the river course scale.

In this study, we intended to learn more about the microbial ecology dynamics in a freshwater environment, verifying the bacterial profile along the length of the river and its relationship with seasonal and environmental changes. To achieve this goal, we performed 16S high-throughput sequencing of 28 samples collected along the Sinos River in two different seasons (summer and winter). Additionally, we performed physicochemical analysis on the same samples to assess environmental indicators. These samples were collected along 173 km of the river, which included the source and the most polluted section of the Sinos River. We showed consistent evidence for the existence of a bacterial seed-bank and its longitudinal and seasonal persistence in the Sinos River. We reemphasize the importance of preserving the source of the river as the main site of the bacterial seed-bank that maintains the identity and ecological integrity of the river.

## Materials and Methods

### Study design, sample collection and physicochemical analysis

**Fig 1:**
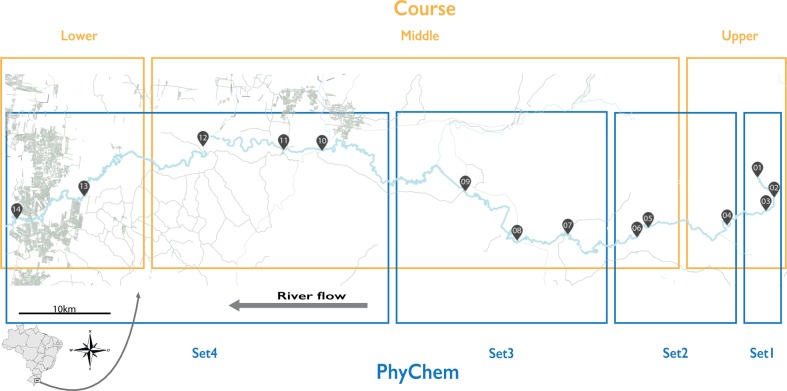
Sinos River course and collection sites. The Sinos River is located in southern Brazil. 14 sampling sites are indicated moving downstream. The Sinos River is indicated by the rectangles separating it into the upper, middle and lower courses. In addition, the physicochemical groupings are indicated by the rectangles in the lower panel as Set1–4.

The water was collected from the epilimnion of the river. We collected 2.1 L of water, of which 100 mL were used for further DNA extraction and 2 L were used for physicochemical analysis. The samples were labeled moving downstream along the Sinos River from the source in Caraá City (sample 1), the most protected region, to São Leopoldo City (sample 14) (Fig. 1), which is one of the most polluted sites on the Sinos River. The collection route comprised 173 km of the river, from which we collected a total of 14 samples in each season. The collection was performed in 2012, in February 14^th^ for summer and August 28^th^ for winter. In both days, we start the collection at 9:00 AM, with samples S01/W01, and follow the river course until samples S14/W14 were collect at 6:23 and 6:47 PM, respectively. Concerning the physicochemical parameters, all samples were analyzed for pH, aluminum, iron, turbidity, conductivity, organic matter, hardness and alkalinity.

### DNA extraction, amplification and sequencing

Total nucleic acids were extracted using the Norgen Biotek Water RNA/DNA Purification Kit with 0.45 μm, following the manufacturer’s protocol. The amplification of 16S was performed as described previously [2,21], using 515F and 806R primers for archaeal and bacterial 16S rDNA V4 region amplification. Each sample were amplified in triplicate, combined and cleaned using the NucleoSpin Gel and PCR Clean-up kit from Macherey-Nagel, following the manufacturer’s protocol. These cleaned amplicons were quantified using the Qubit dsDNA HS Assay. The final sample for sequencing was created by combining equimolar ratios of the amplicons from the individual samples. The sample was sent to FASTERIS (http://fasteris.com) for sequencing in MiSeq, using V2 kit 2x250.

### Filtering quality and sequence analysis

The pair-end reads from MiSeq were filtered to remove any adapter and primers sequences using a customized python script, where we allowed up one mismatch to retain the sequence for downstream analysis. Then, a quality filter was applied to eliminate reads with one or more indeterminate bases, “N”, and truncated sequences with two or more consecutive bases with quality scores below to Q30. The remaining read pairs were assembled using Pandaseq [22]. The downstream analysis was performed using QIIME version 1.7.0 [23] and Greengenes version 13.8 [24] as a reference database. The OTU Picking was performed using an open reference OTU-picking pipeline against the 97% identity of the preclustered Greengenes using Uclust [25], followed by testing the removing of OTUs with representation of 2, 3, 4, 5, 10, 20, 40, 80 and 160 reads (S1 Table). We also evaluated the alpha-diversity through Faith’s phylogenetic diversity [26], the Chao1 species richness index [27] and OTU richness (numbers of OTU). Furthermore, we calculated the beta-diversity using UniFrac [28,29], which is a metric distance for comparing bacterial communities among samples. To test the correlation among the UniFrac matrix and the geographical distances, we performed a Mantel test with compare_distances_matrix in QIIME pipeline. To achieve statistical confidence for the sample categorization (Season, PhyChem and Location), we performed the adonis and ANOSIM tests using the vegan package [30]. We used SourceTracker [31], which is a Bayesian approach, to infer to what extent the microbial community that forms a set of samples (source) could explain the existing diversity of organisms in another set of samples (sink).

## Results

The Sinos River microbiome samples were categorized into four different groups, based on the following environmental data (Table 1): i) Location, the collecting sites of the samples; ii) Course, the course sections of the river; iii) PhyChem, groups of physicochemical features; and iv) Season, the season in which the samples were collected (summer and winter). To define groups of samples based on PhyChem, we tested a series of groupings using ANOVA. Then, we used the four groups of samples with the lowest standard deviations based on the physicochemical similarities (Table 1, Fig. 1 and S1 Fig.).

**Table 1:** Description of local of samples collections with GPS coordination, samples names and classifications (Set and PhyChem) and absolute and relative distance in the water course.

Location	Course	GPS		PhyChem	Distance (km)		Sample Code	
		Latitude	Longitude		Absolute	Relative	Summer	Winter
1	Upper	9°42′24.13"S	50°17′29.37"W	Set1	0	0	S01	W01
2	Upper	29°44′20.97"S	50°16′17.94"W	Set1	4.9	4.9	S02	W02
3	Upper	29°44′58.32"S	50°16′59.35"W	Set1	6.76	1.86	S03	W03
4	Upper	29°45′45.20"S	50°19′39.06"W	Set2	13.45	6.69	S04	W04
5	Middle	29°45′51.75"S	50°25′39.40"W	Set2	27.58	14.13	S05	W05
6	Middle	29°46′34.45"S	50°26′0.65"W	Set2	29.17	1.59	S06	W06
7	Middle	29°46′22.61"S	50°31′4.50"W	Set3	44.43	15.26	S07	W07
8	Middle	29°46′41.90"S	50°34′7.05"W	Set3	53.33	8.9	S08	W08
9	Middle	29°43′32.23"S	50°38′12.44"W	Set3	70.45	17.12	S09	W09
10	Middle	29°41′12.45"S	50°48′22.25"W	Set4	109.77	39.32	S10	W10
11	Middle	29°41′10.36"S	50°51′2.96"W	Set4	114.65	4.88	S11	W11
12	Middle	29°41′28.52"S	50°56′30.08"W	Set4	132.09	17.44	S12	W12
13	Lower	29°43′52.72"S	51° 5′1.14"W	Set4	157.17	25.08	S13	W13
14	Lower	29°45′36.25"S	51° 9′59.99"W	Set4	173.25	16.08	S14	W14

### Microbiome diversity and seed-bank persistence longitudinally and across seasons

**Fig 2:**
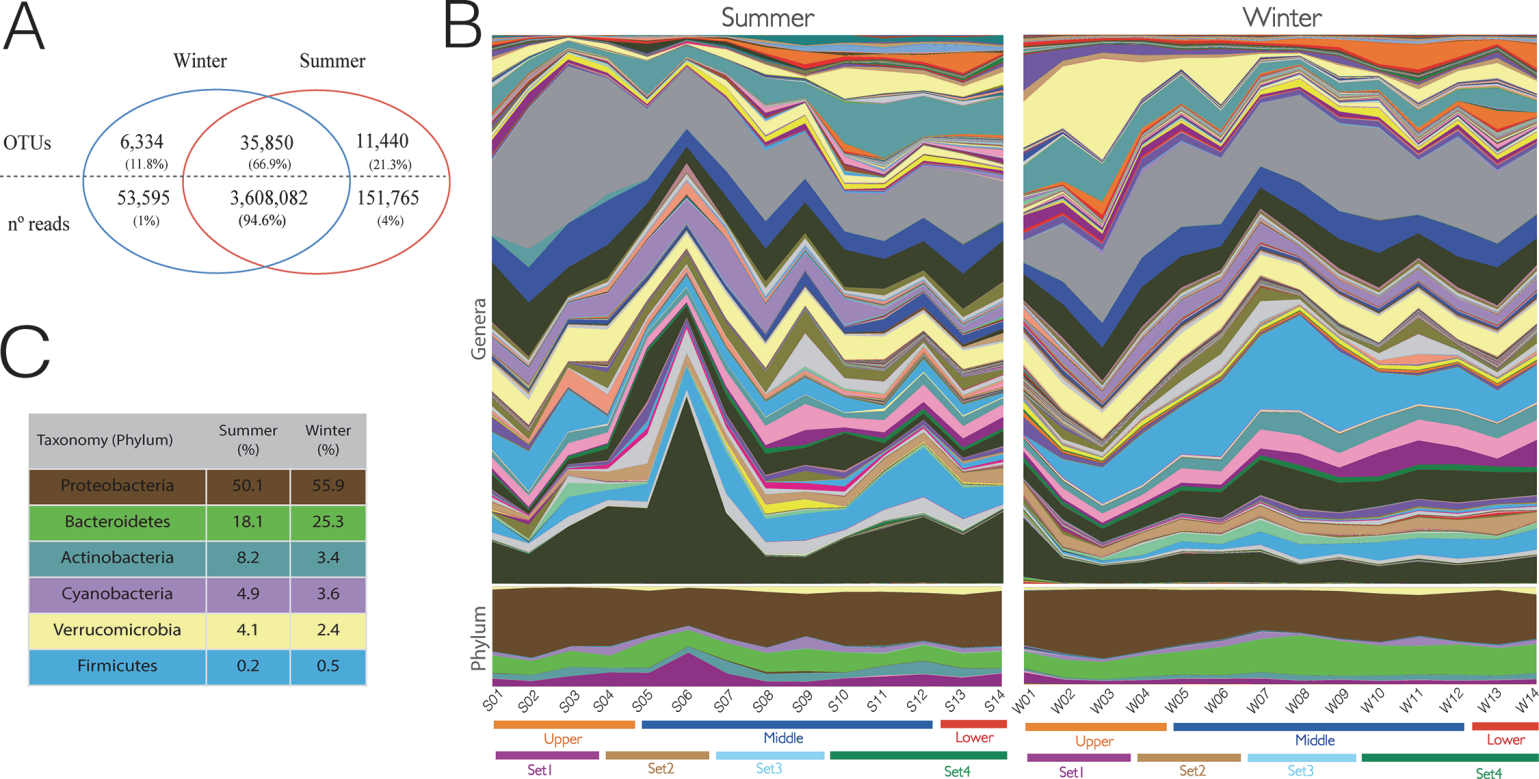
Sinos River OTUs analysis and classification. **(A)** The Venn diagram shows the number of OTUs present in both seasons combined and each season. Blue circles indicate winter, and red indicates summer. In addition, the total number of reads belonging to the OTUs and their correspondent percentage in the total are shown. **(B)** Table of the most abundant phyla found in the analysis, with their respective proportions in the summer and winter samples. **(C)** Profile showing the proportion of phyla for each sample in summer (S01-S14) and winter (W01-W14). The profile also represents the proportion grouped along the course of the Sinos River (upper, middle and lower) and PhyChem analysis (Set1–4). Colored profiles correspond to the most abundant phyla presented in (B).

Through sequencing barcoded amplicons from the 16S V4 region in the MiSeq Illumina platform, we generated a total of 5,790,065 pair-end reads for the 28 samples, which passed through rigorous quality control, classification through the OTU open reference picking process and OTU represented by less than 5 reads was discarded (S1 Table). This analysis resulted in 53,624 OTUs, which 35,850 OTUs (66.9%) were found in at least one sample from each season, representing 94.6% of all classified reads (Fig. 2A). These results strongly suggest that the bacterioplankton of the Sinos River are highly homogeneous in composition, exhibiting a seed-bank of bacteria in the river source (S01 and W01). Additionally, we found 6,334 OTUs (11.8%) restricted to winter and 11,440 (21.3%) OTUs restricted to summer samples. These OTUs found exclusively in summer and winter represent 4% and 1.4%, respectively, of all classified reads. Additionally, about of 93.8% of the reads classified in all samples refer to 28,260 OTU present at samples S01 and W01, from the river’s source.

**Fig 3:**
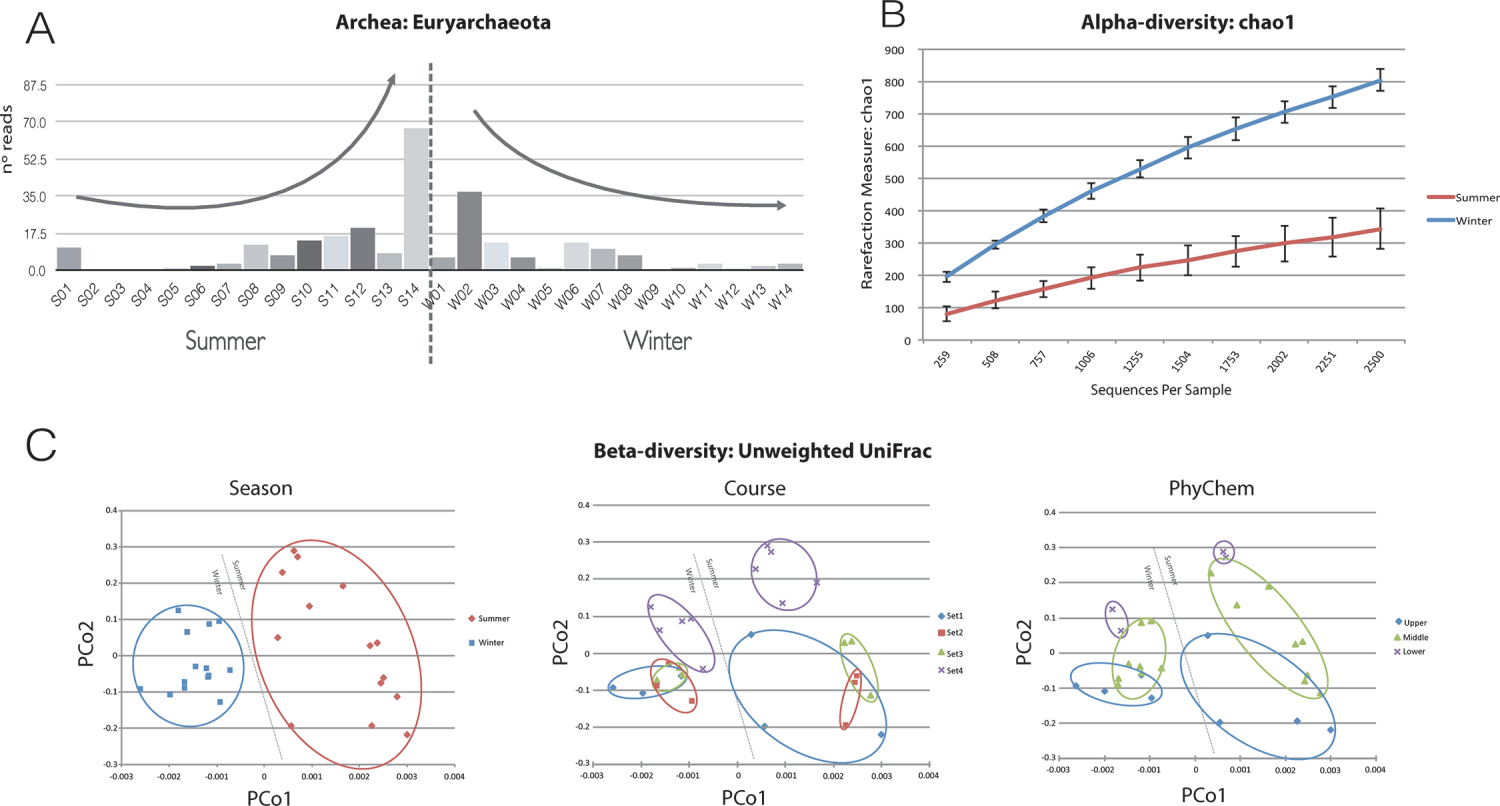
Archaeal and bacterial diversities. **(A)** Proportion of reads classified as Euryarchaeota from Domain Archaea. In summer, this phylum increased its representation with increasing eutrophication, and in winter, there is a gradual longitudinal decrease in the abundance of this phylum. **(B)** Alpha-diversity results using Chao1 for summer and winter. The results show a tendency of greater homogeneity and Chao1 values in the winter than in the summer. **(C)** Beta-diversity results, using unweighted Unifrac. The analyses were performed independently for the sample groups representing seasons, course and PhyChem classification.

We found a total of 4 bacteria phyla, with Proteobacteria, Bacteroidetes, Actinobacteria, Cyanobacteria, Verrucomicrobia and Firmicutes as the most representative groups found in all samples (Fig. 2B, 2C and S2 Table), representing a total of 85.6% and 91.1% of all classified reads for the summer and winter samples, respectively. At the family taxonomic level, we found Comamonadaceae, ACK-M1, Rhodobacteraceae, Chitinophagaceae and Flavobacteriaceae as the five most representative taxa for both, summer and winter samples (S2 Fig.). The Pseudomonadaceae is the family that presented the higher discrepancy between summer and winter, representing 0.5% and 2.7%, respectively. For Domain Archaea, we found 85 OTUs classified as Parvarchaeota, Crenarchaeota and Euryarchaeota, represented by 1,777 reads in all samples combined. Interestingly, the phylum Euryarchaeota showed an inverted trend between summer and winter (Fig. 3A). We found an increasing number of reads moving downstream, with a peak of read abundance in the S14 summer samples. In contrast, we found a decreasing abundance moving downstream of Euryarchaeota in winter, with the W02 sample having higher numbers of reads and the abundance gradually decreasing to W14.

As consistent evidence was found for a primary core group of bacteria in samples S01 and W02, and as its spread downstream was evident as well, we seek to measure the seasonal persistence of this pattern using SourceTracker [31], a Bayesian approach. The proportion of the OTUs found in the winter that could be explained by the persistence of summer OTUs was measured. Then, we tested the samples from summer as the source and winter as the sink. The samples were also analyzed in groups by their Course and PhyChem classifications (S3 Fig.) to test for longitudinal differences and to assess the structure and origins of the populations. On average, approximately 79% of the microbial composition from the winter samples could be explained by the summer samples, using both the Season and PhyChem categorizations. Interestingly, approximately 6% of winter samples W01-W09 could be explained by the bacterial composition and structure of the summer samples from the lower or set4 groups.

### Alpha and beta-diversity correlations with seasons, longitudinal distance and environmental parameters

Using Chao1 richness, we tested the alpha-diversity for the Season, PhyChem and Course groups. For Season, we found higher Chao1 richness values in the winter samples than in the summer samples (Fig. 3B). For the PhyChem and Course categorizations (S4 Fig.), the same tendency was observed in both groups, with the samples from the most impacted regions of the Sinos River (Set4 and the lower group, respectively) having higher alpha-diversity than samples from the less impacted regions. Additionally, we verified the alpha-diversity for each season separately. For the winter samples, a modest trend was observed of higher Chao1 values in the samples from the PhyChem and Course classifications for set4 and the lower group, respectively. However, in the summer samples, this pattern was more accentuated. The samples from set4 and the lower group showed higher values of alpha-diversity (S5 Fig.).

The beta-diversity calculated for all samples together using unweighted UniFrac (Fig. 3C and S7 Fig.) visually suggests a correlation among the seasonal classifications. Adonis and ANOSIM were applied to assess the statistical significance between the unweighted UniFrac dissimilarity matrix and each of the categories used for the four different classifications (Location, Season, Course and PhyChem) (Table 2). The statistical analysis indicates that the categories for Season, Course and PhyChem were significant. Nevertheless, seasonal classification showed a better correlation with the UniFrac results, whereas Location was not significantly correlated with them. To test the correlation between the geographic distance and UniFrac dissimilarity, we performed a mantel test implemented in the QIIME pipeline. A lower correlation between geographic distance and unweighted UniFrac (r = 0.28, p-value = 0.003) was obtained. Furthermore, we calculated the Unifrac dissimilarity for the samples from both seasons separately. These results showed that, independent of the season, the samples from the river source (S01 and W01) have significant dissimilarity from the other sections of the river (S6 Fig.).

**Table 2:** Results for Adonis and ANOSIM comparing unweighted Unifrac matrix with four categorizations (Course, PhyChem, Location and Season).

	All Samples				Summer				Winter			
	Adonis		ANOSIM		Adonis		ANOSIM		Adonis		ANOSIM	
	R2	pvalue	R	pvalue	R2	pvalue	R	pvalue	R2	pvalue	R	pvalue
Course	0.099	**0.005**	0.294	**0.004**	0.21	**0.003**	0.441	**0.009**	0.198	**0.005**	0.503	**0.003**
PhyChem	0.148	**0.001**	0.239	**0.003**	0.309	**0.001**	0.254	**0.036**	0.279	**0.003**	0.413	**0.003**
Location	0.469	0.768	-0.055	0.684	-	-	-	-	-	-	-	-
Season	0.098	**0.001**	0.472	**0.001**	-	-	-	-	-	-	-	-

The results with p<0.05 are bolded.

## Discussion

Microorganisms are the most abundant and ubiquitous organisms on our planet. In aquatic ecosystems, they play a critical role in almost all environmental and ecological processes. By applying deep sequencing of the 16S V4 region, we performed microbiome surveillance, analyzing the microbiome of the Sinos River at the course scale using high-throughput methods. We showed that there is a core group of OTUs, representing 94.6% of all sequenced reads that was present in both seasons and in samples from the source throughout the length of the river.

Our results strongly suggest the longitudinal persistence of a bacterial core group and the maintenance of a seed-bank in the river source across seasons. This seed-bank of OTUs in our data represent about 93.8% of all reads sequenced for all samples. The maintenance of a bacterial seed-bank had been previously demonstrated for marine ecosystems [8,9], and more recently, the presence of a seed-bank in the Mississippi River was observed through changes in the relative abundance at different sites along the river [17].

The S01 and W01 samples were collected from a shallow water site, with rapid flow transporting particles from the substrate. Previous work in artic freshwater has shown that the microbial diversity is structured by inoculation of bacteria from the soil [13] with continued transported along the course of the river [16,32]. These scenarios are consistent with the OTU profiles and our SourceTracker results, which illustrate a high proportion of OTUs shared among samples from summer and winter and along the length of the Sinos River. In the SourceTracker results, we found approximately 6% of the summer Set4 was related to the winter samples W01-W09, suggesting that some OTUs in the river source, unstructured and in lower abundances, could increase in abundance 70 Km downstream, when the environment is quite different from the river source. Additionally, the SourceTracker results indicates that summer Set2 was the least represented group in all of the winter samples. This unexpressed contribution of summer Set2 could be explained through the hypothesis that this is a transitory region, without a structured population, in comparison with the other groups.

The alpha-diversity results for the two seasons suggest some biodiversity decrease in the summer compared with the winter. This pattern has been described in previous studies of marine microbiome dynamics, and this difference is not attributed to the richness of each community; instead, it was suggested to be related to the uniformity of the taxa [7,8]. Furthermore, we found a more pronounced alpha-diversity value for the summer samples from set4 (PhyChem) and the lower (Course) section, which are more disturbed areas, than from the upstream section, closest to the source of the river. Interestingly, when we analyzed the alpha-diversity of the summer and winter samples separately, the winter samples had similar alpha-diversity throughout the length of the river. This could also suggest more homogenous bacterial diversity along the Sinos River during winter. In contrast, there is an increase in the alpha-diversity values in the summer in the samples from the more polluted river regions (Set4/Lower), suggesting that these particular locations have increased bacterial diversity in summer.

The analysis of the beta-diversity results using unweighted Unifrac and microbiome profile analysis suggests a seasonal community structure, with more community homogeneity in the winter samples than in summer samples. Previous studies have shown that some microbial communities can be resilient to environmental disturbances [1,14]. The winter in the southern region of Brazil is marked by increased rainfall, increasing the river flow and homogenizing the river. With its capability for resilience, these processes could explain the homogeneity of the microbial community that we observed in winter. In general, we were not able to establish a strong correlation between the bacterioplankton profile and isolated physicochemical parameter; however, we observed that bacterioplankton responds to the environment as a whole. When we use categorizations from the physicochemical profiles (PhyChem) or geo-hydrographic features (Course), we found statistical correlations for the composition and structure of the bacterial communities. Previously work has been reported phyla usually found in lake freshwater bacterial community [33]. This set include phyla like Proteobacteria, Actinobacteria, Bacteroidetes, Cyanobacteria and Verrucomicrobia. Actinobacteria as been described as the most common phylum in the lake freshwater epilimnion and is responsive to pH. In our results we found about 8.0% and 3.4% of Actinobacteria in the summer and winter samples, and samples from summer haves, in average, a higher pH, than winter samples. The ACK-M1 family, from Actinobacteria phylum, is usually more prevalent in higher pH. In our results, ACK-M1 represents 5% of identified family in summer’s samples, and about 1.2% in winter’s one. This was the most strong evidence of environmental microbial driven related with an isolated physicochemical parameter.

In conclusion, our analysis of the Brazilian Sinos River microbiome support previous ideas that microbial populations are maintained by a core of OTUs from the seed-bank that persist longitudinally and seasonally. So far, despite the differences among seasons and the river’s course environment, we noticed that the main core of bacterial diversity is maintained, whereas population structure is divergent along the river’s course and seasons. One hypothesis to explain these observations is that the river’s source is continuously restoring the microbial diversity found along the river. These findings reinforce the importance of the preservation of river sources as one of the ecological keys for the recovery and maintenance of impacted rivers.

## Supporting Information

S1 Fig Grouping of the samples for the physicochemical parameters. The water collected was analyzed for aluminum, iron, turbidity, hardness, organic matter, conductivity and alkalinity. The statistical analysis was performed using one-way ANOVA with Ducan test considering p<0.05. The letters represent groups that are statistically significant.
(PDF)

S2 Fig Profile of bacterioplankton at the family taxonomy level. Area chart of results from the otu_pickup pipeline representing the family levels or the lowest level that was possible to classify.
(PDF)

S3 Fig SourceTracker results for the Course and PhyChem analyses. This analysis demonstrates the proportion of the winter bacterioplankton that could be explained by samples from the summer. The Course results are in the upper panel, separated by the course categorization into upper, middle and lower. The bottom panel shows the results for the PhyChem analysis for each winter sample in the four different physicochemical sets (Set1–4). The results could be related to the middle panel illustrating the Sinos River and the sampling points. The colored bars for each winter sample represent the proportion of the bacterial structured populations that was present in the summer in that course of the river, based on the Course and PhyChem categorizations.
(PDF)

S4 Fig Alpha-diversity with seasonal samples combined. Plot of the alpha-diversity results for the summer and winter samples combined by the PhyChem and Course groups.
(PDF)

S5 Fig Alpha-diversity in winter and summer. Plot representing the independent analysis of the samples for both seasons, summer and winter, by the Course and PhyChem categorizations.
(PDF)

S6 Fig Beta-diversity using unweighted UniFrac. Principal Coordinate Analysis showing the correlations for the individual samples in both seasons, summer and winter, by the Sinos River PhyChem and Course categorizations.
(PDF)

S7 Fig Beta-diversity using unweighted UniFrac for representation cutoff. Principal Coordinate Analysis showing the correlations for the individual samples in both seasons, summer and winter, by the Sinos River PhyChem and Course categorizations applied to OTU cutoff of 2, 5 and 160 reads.
(PDF)

S1 Table Quantitative results of OUT picking process and remained reads and identified OUT for each read coverage cutoff. Results for total amount of reads and number of recovered OTU after application of each minimum read coverage cutoff.
(XLSX)

S2 Table OTUs identified in the Sinos River. GreenGene ID and taxonomy classification are presented for each sample for summer (S) and winter (W) along with the number of reads for the OTUs identified.
(XLSX)

S3 Table SourceTrack results for the Course and PhyChem classification. The columns represent the set of samples for PhyChem (Set1–4) and Course (upper, middle and lower) used as the potential source. The rows represent the samples from winter, which were used as the sink, to determine the percent similarity in relation to the summer samples. Unknown values indicate the percentage of the differences in the bacterial composition and structure that are not present in the summer samples.
(XLSX)
