# Supplementary material for: The Source of the River as a Nursery for Microbial Diversity
Source: PLoS One. 2015 Mar 24;10(3):e0120608. doi: 10.1371/journal.pone.0120608 (PMC4372583; doi:10.1371/journal.pone.0120608)
Supplement: S1 Fig — The water collected was analyzed for aluminum, iron, turbidity, hardness, organic matter, conductivity and alkalinity. The statistical analysis was performed using one-way ANOVA with Ducan test considering p<0.05. The letters represent groups that are statistically significant. (PDF) [file pone.0120608.s001.pdf]

S1 Fig.

# PhyChem

## Physicochemical parameters

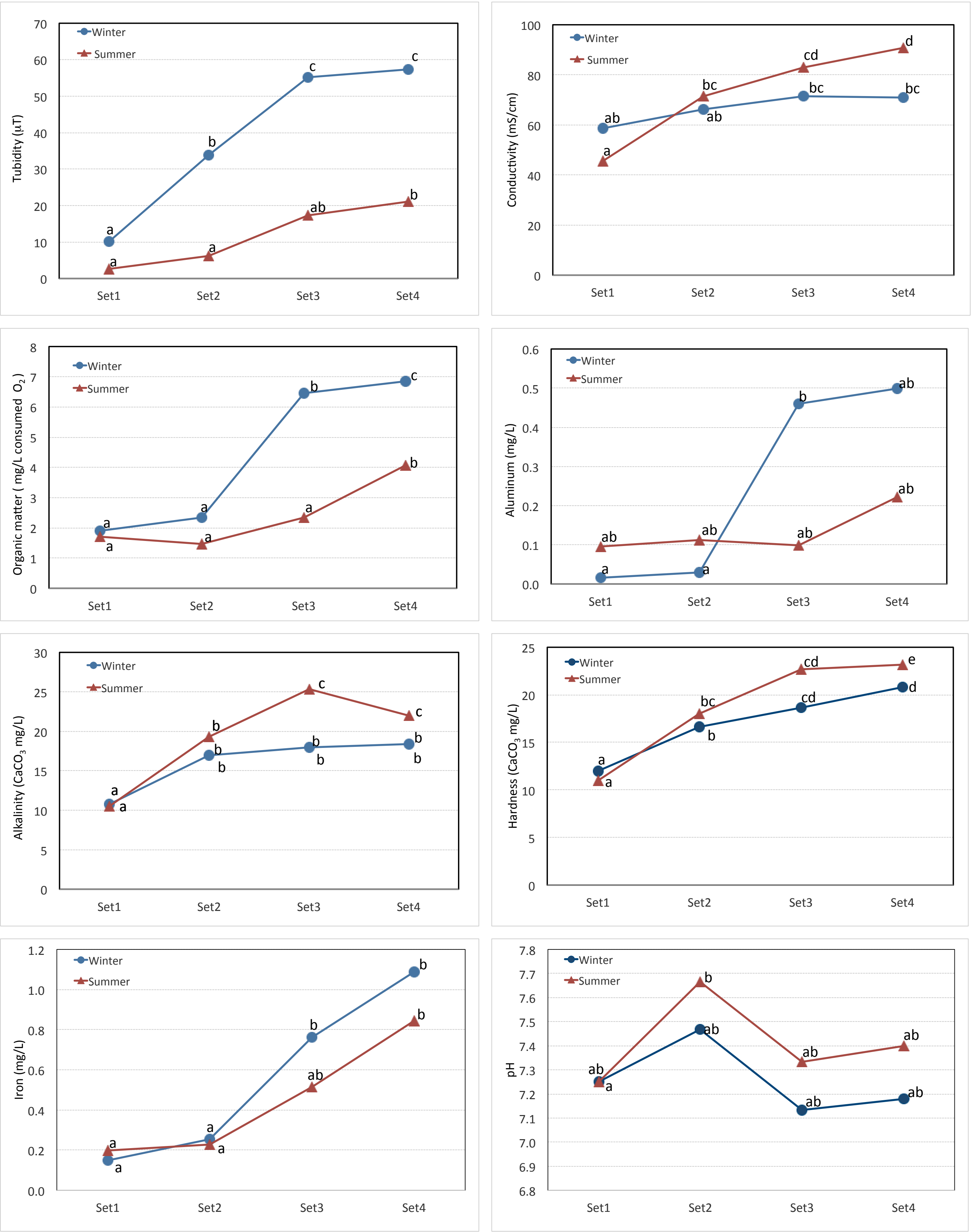

S2 Fig.

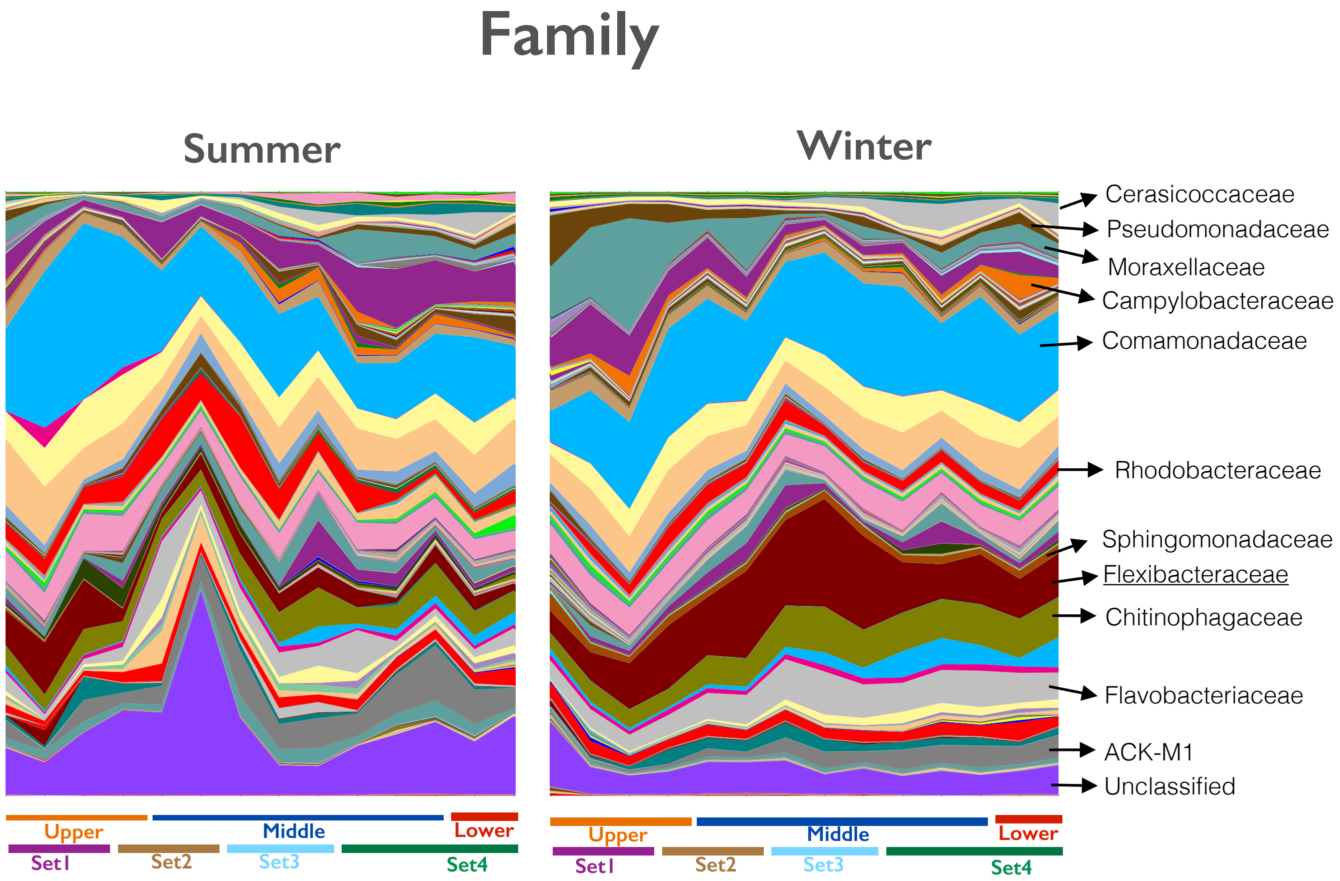

Family
Summer
Winter
Upper
Middle
Lower
Set1
Set2
Set3
Set4
Cerasicoccaceae
Pseudomonadaceae
Moraxellaceae
Campylobacteraceae
Comamonadaceae
Rhodobacteraceae
Sphingomonadaceae
Flexibacteraceae
Chitinophagaceae
Flavobacteriaceae
ACK-M1
Unclassified

S3 Fig.

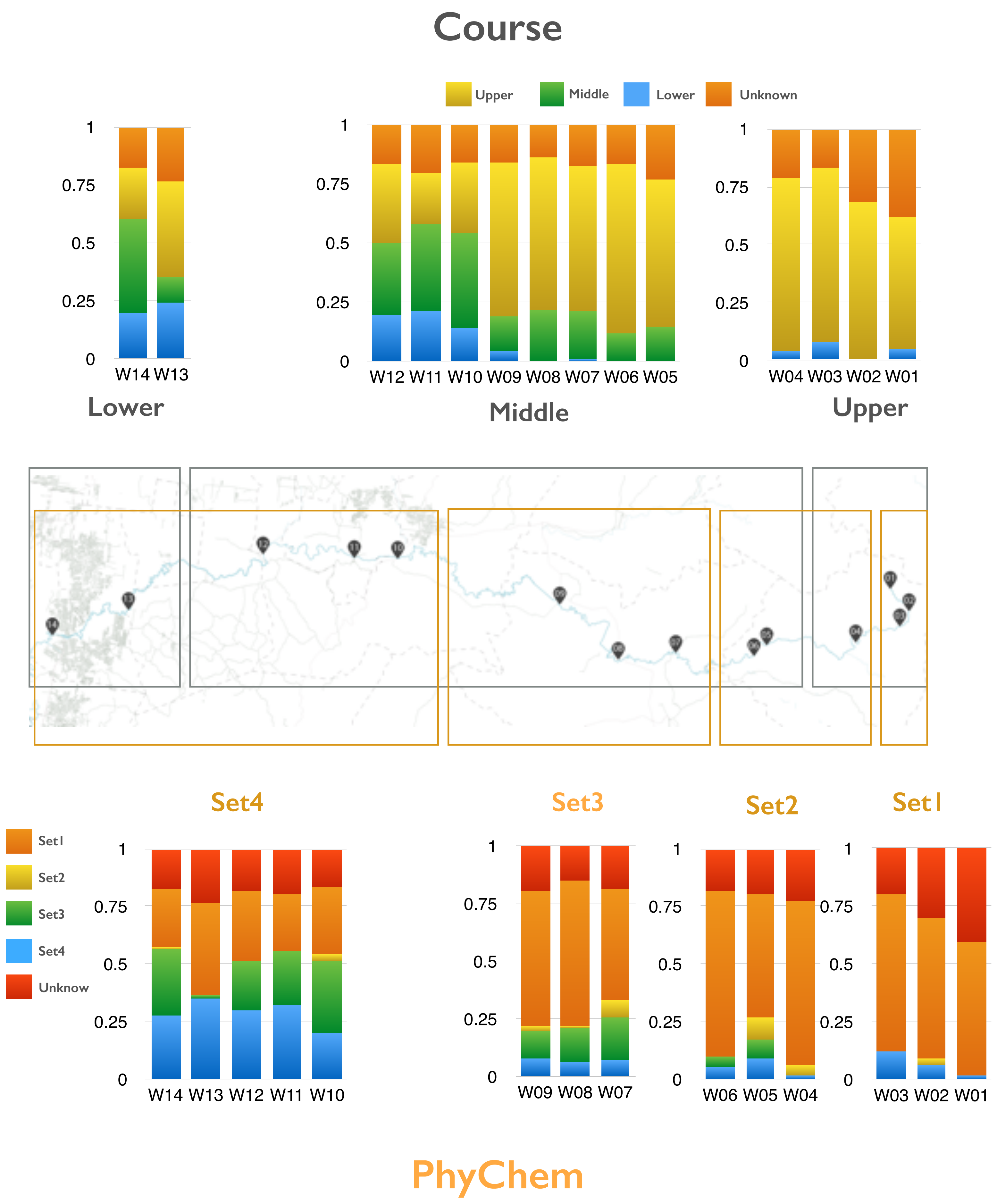
Course
Upper
Middle
Lower
Unknown
1
0.75
0.5
0.25
0
W14 W13
Lower
W12 W11 W10 W09 W08 W07 W06 W05
Middle
W04 W03 W02 W01
Upper
Set4
Set3
Set2
Set1
Set1
Set2
Set3
Set4
Unknow
W14 W13 W12 W11 W10
W09 W08 W07
W06 W05 W04
W03 W02 W01
PhyChem

S4 Fig.

# Alpha-diversity

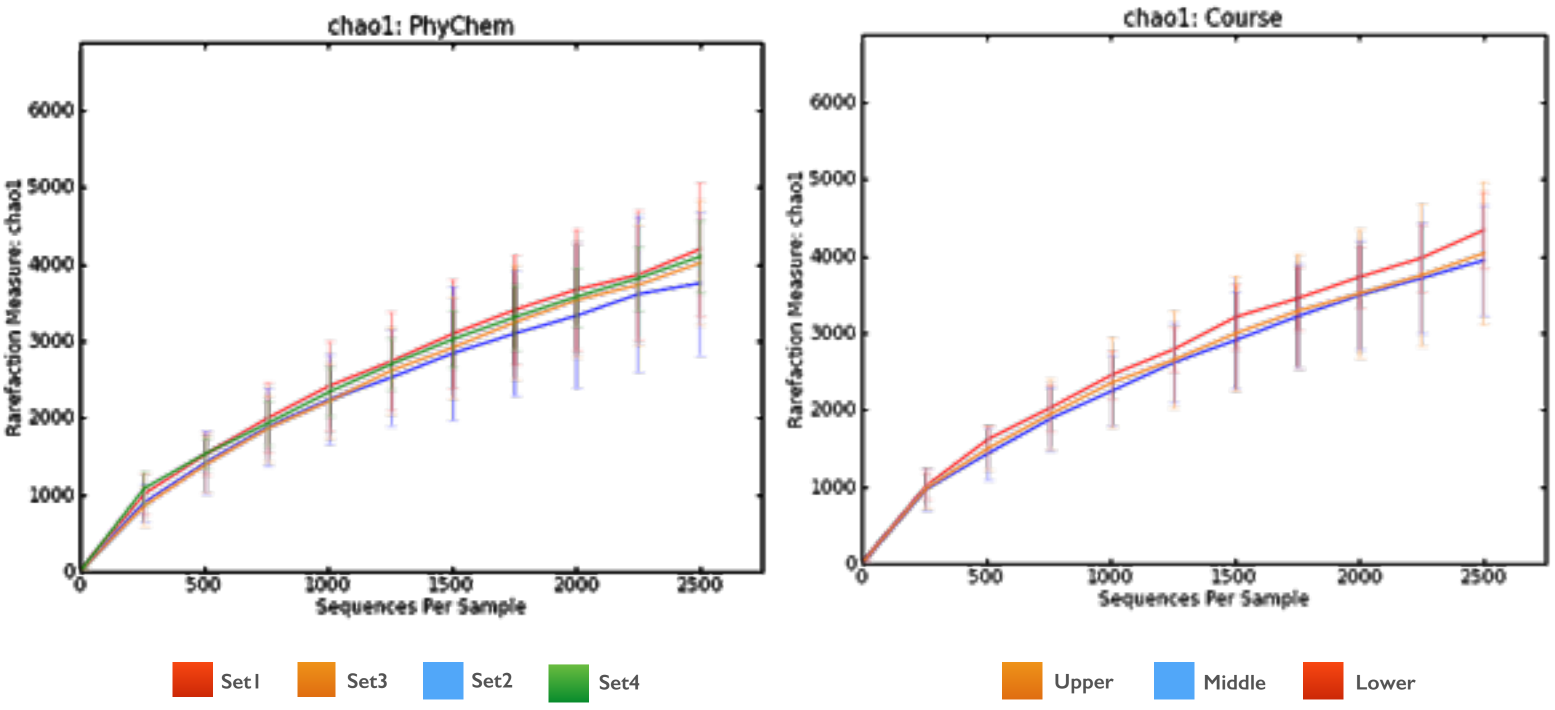

S5 Fig.

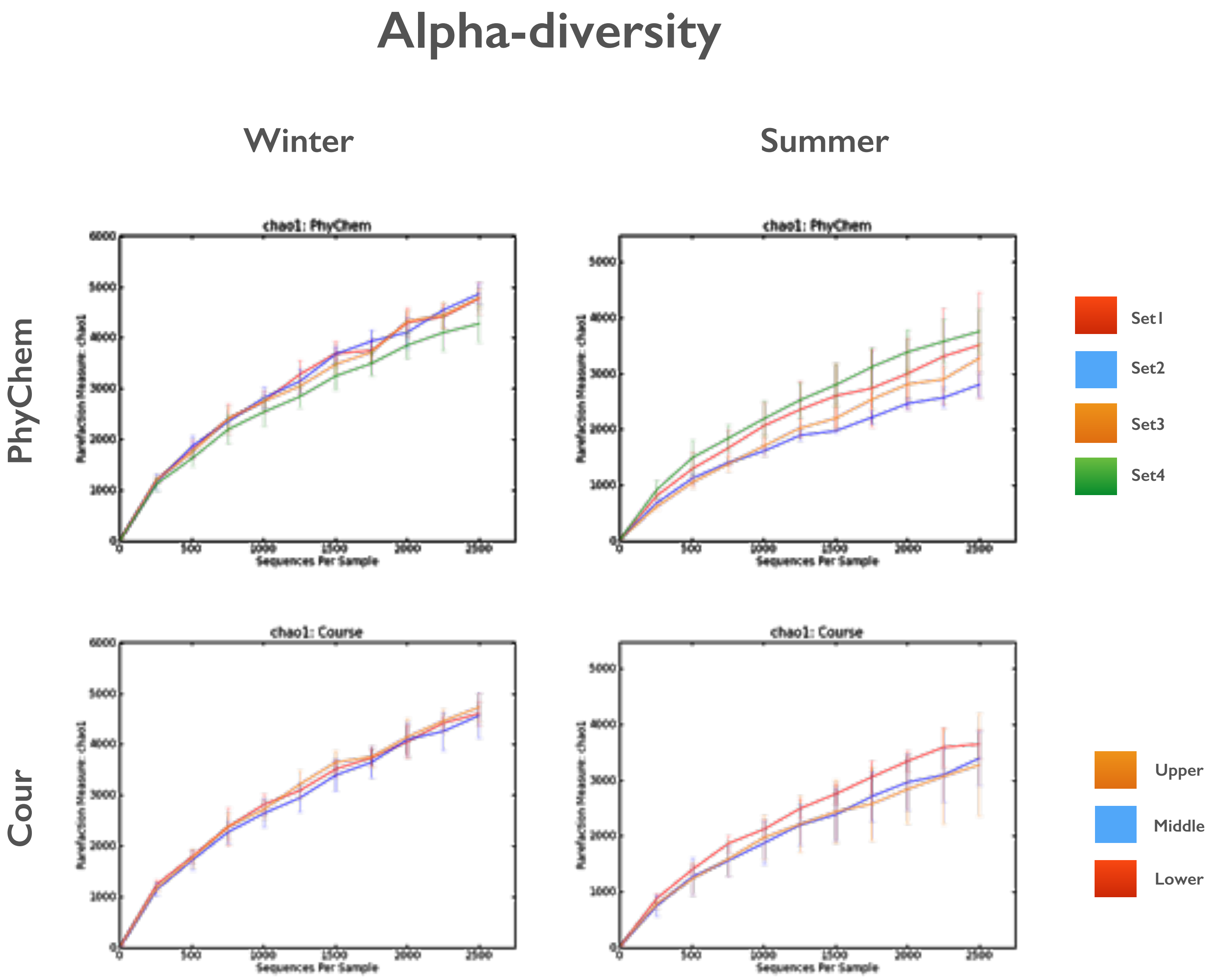

Alpha-diversity
Winter
Summer
PhyChem
Cour
chao1: PhyChem
chao1: PhyChem
chao1: Course
chao1: Course
Sequences Per Sample
Rarefaction Measure: chao1
Set1
Set2
Set3
Set4
Upper
Middle
Lower

S6 Fig.

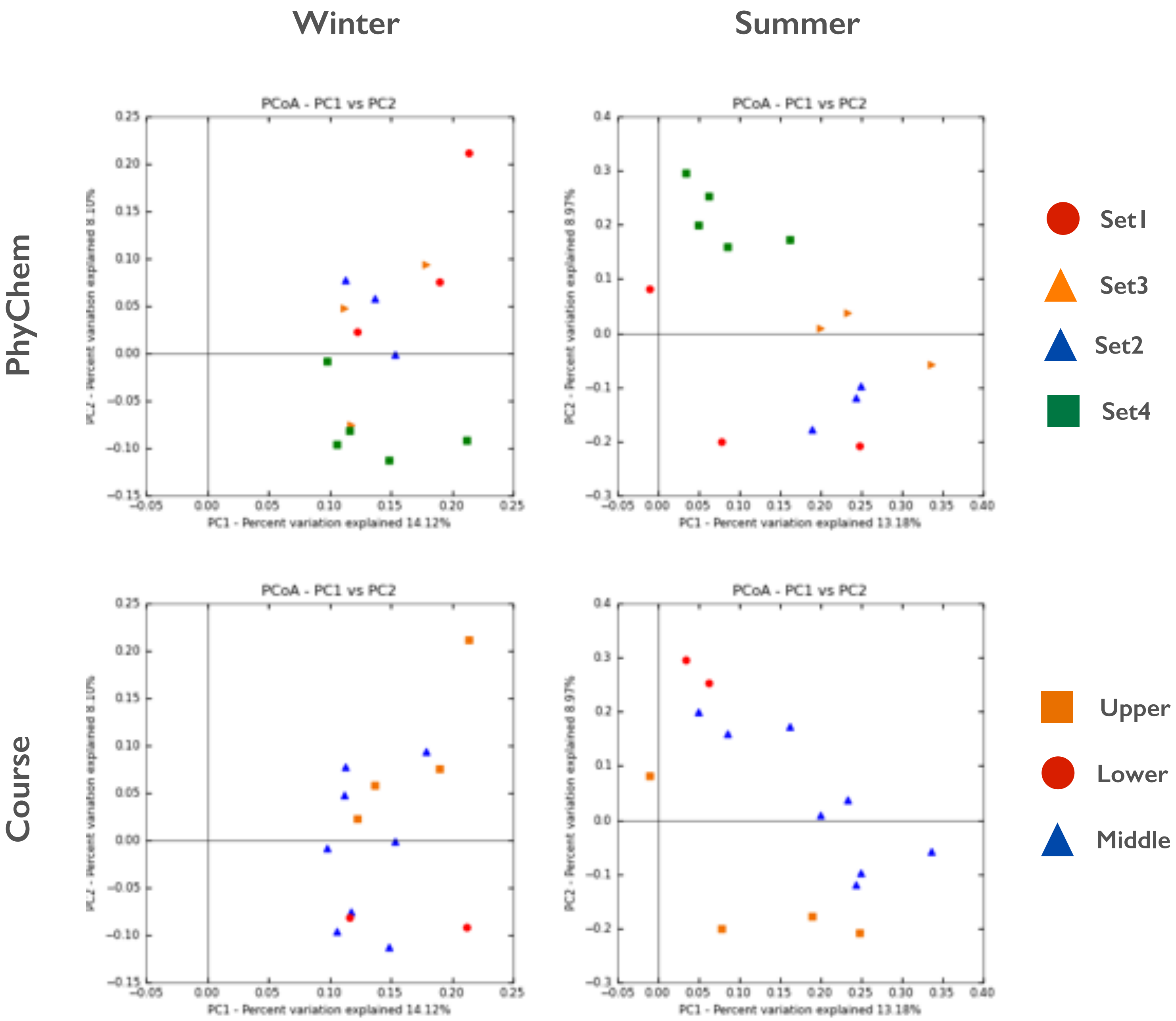
Winter
Summer
PhyChem
Course
PCoA - PC1 vs PC2
PC1 - Percent variation explained 14.12%
PC1 - Percent variation explained 13.18%
PC2 - Percent variation explained 8.97%
Set1
Set3
Set2
Set4
Upper
Lower
Middle

S7 Fig.

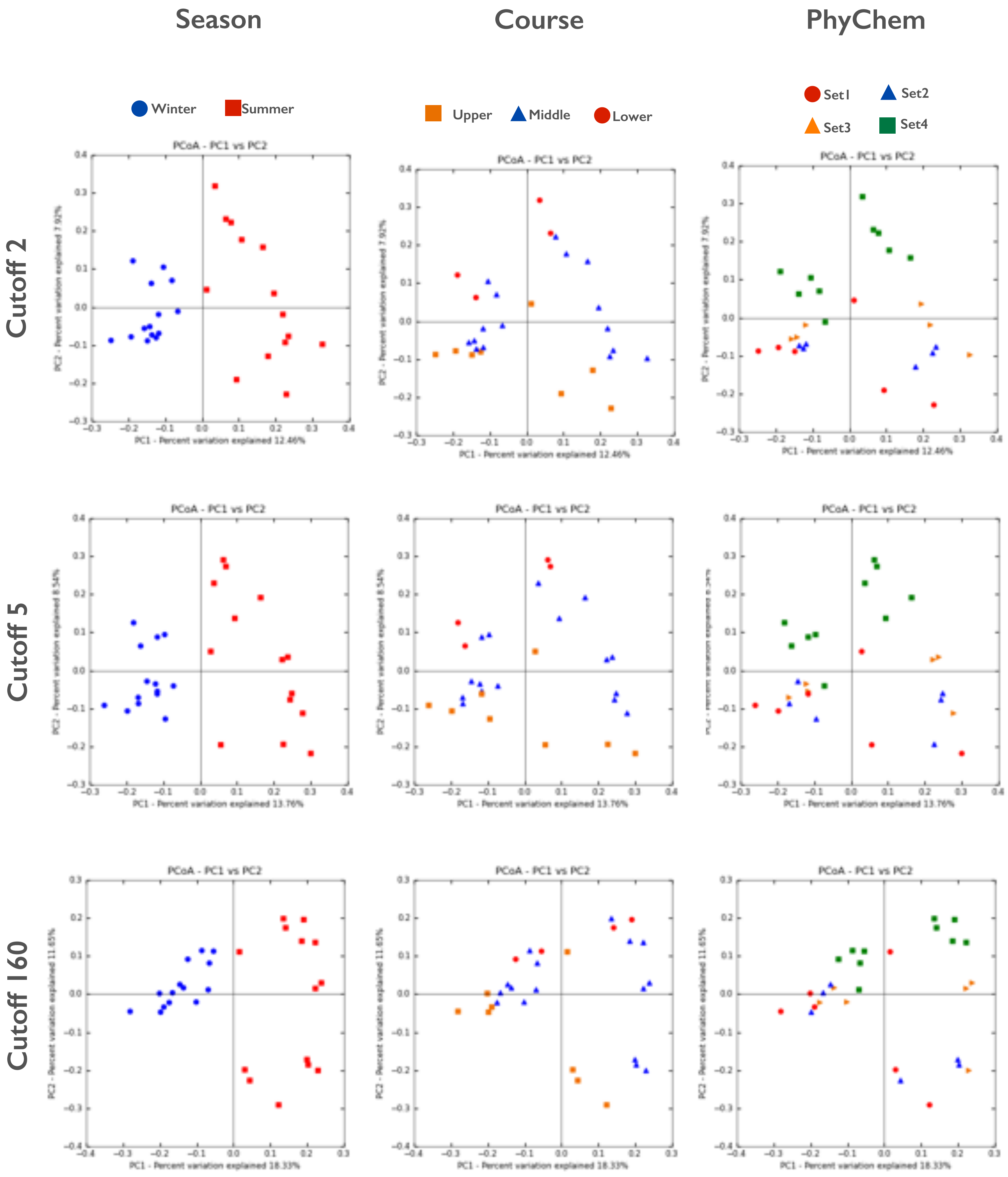
Season
Course
PhyChem
Winter
Summer
Upper
Middle
Lower
Set1
Set2
Set3
Set4
Cutoff 2
Cutoff 5
Cutoff 160
PCoA - PC1 vs PC2
PC1 - Percent variation explained 12.46%
PC2 - Percent variation explained 7.92%
PC1 - Percent variation explained 13.76%
PC2 - Percent variation explained 8.54%
PC1 - Percent variation explained 18.33%
PC2 - Percent variation explained 11.65%
