# Supplementary figures and images for: The Source of the River as a Nursery for Microbial Diversity
Source: PLoS One. 2015 Mar 24;10(3):e0120608. doi: 10.1371/journal.pone.0120608 (PMC4372583; doi:10.1371/journal.pone.0120608)

S2 Fig.

# Family

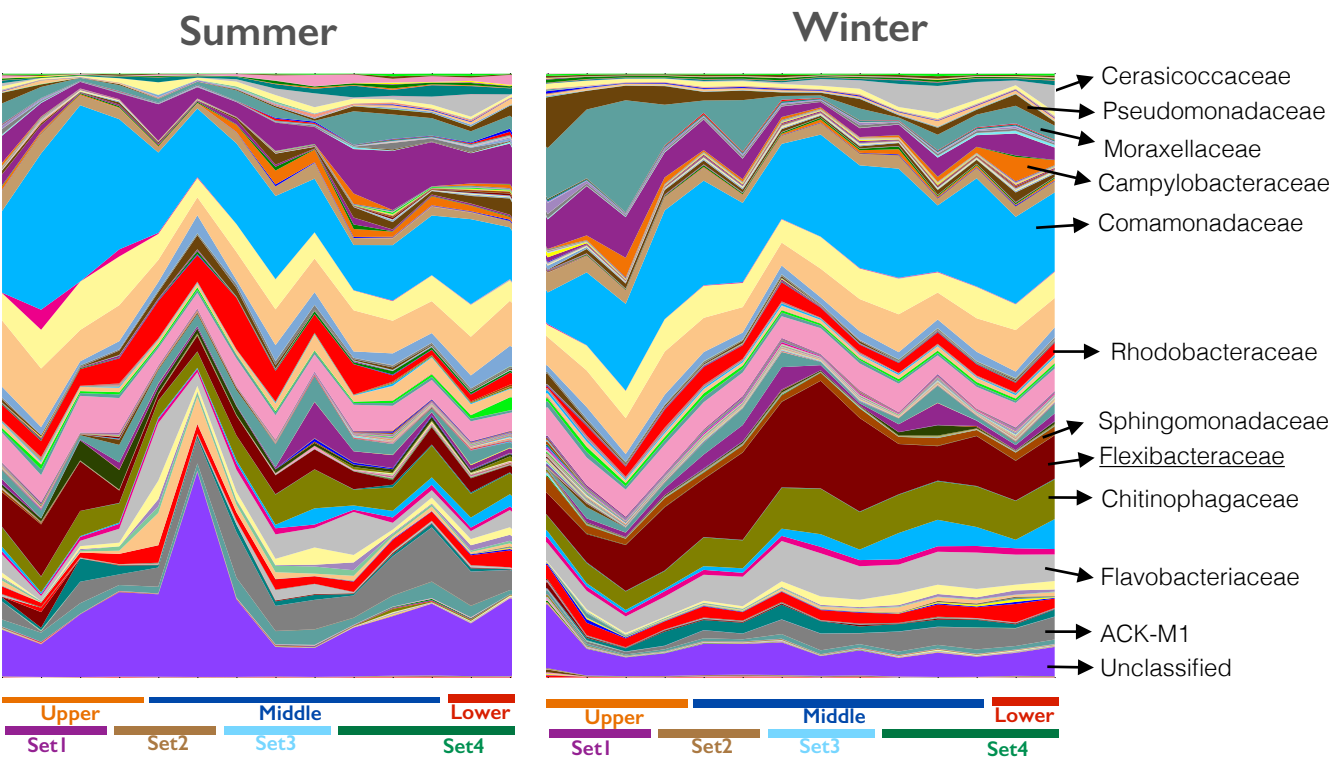

Supplement: S2 Fig — Area chart of results from the otu_pickup pipeline representing the family levels or the lowest level that was possible to classify. (PDF) [file pone.0120608.s002.pdf]

S3 Fig.

## Course

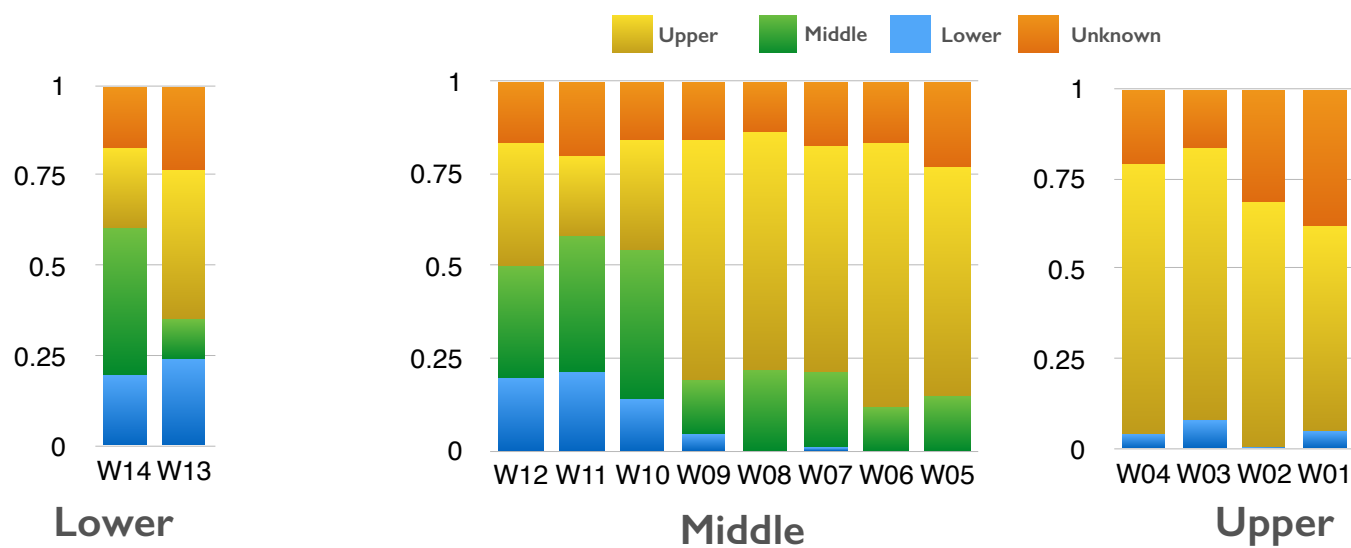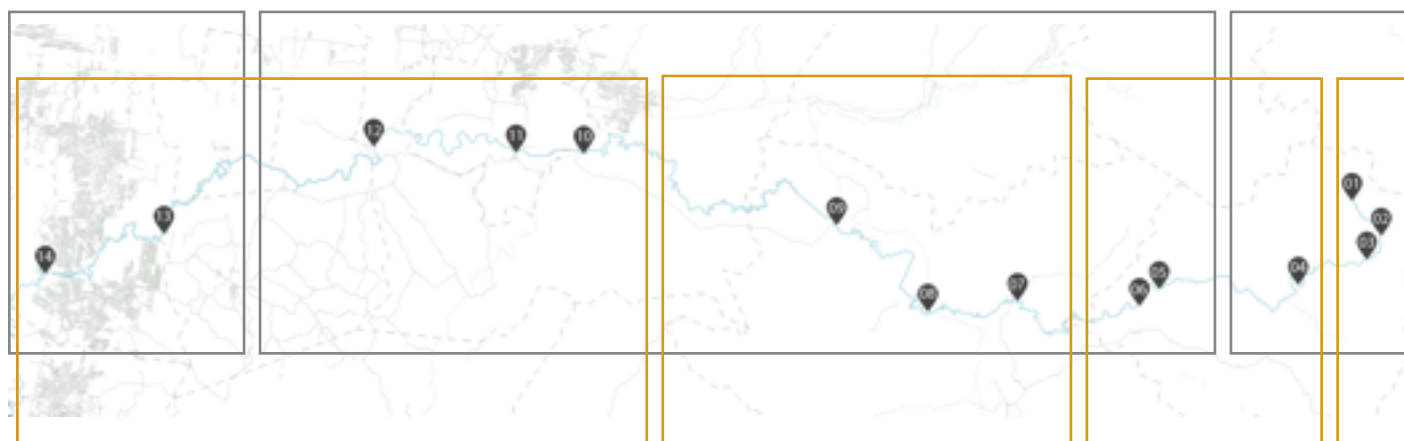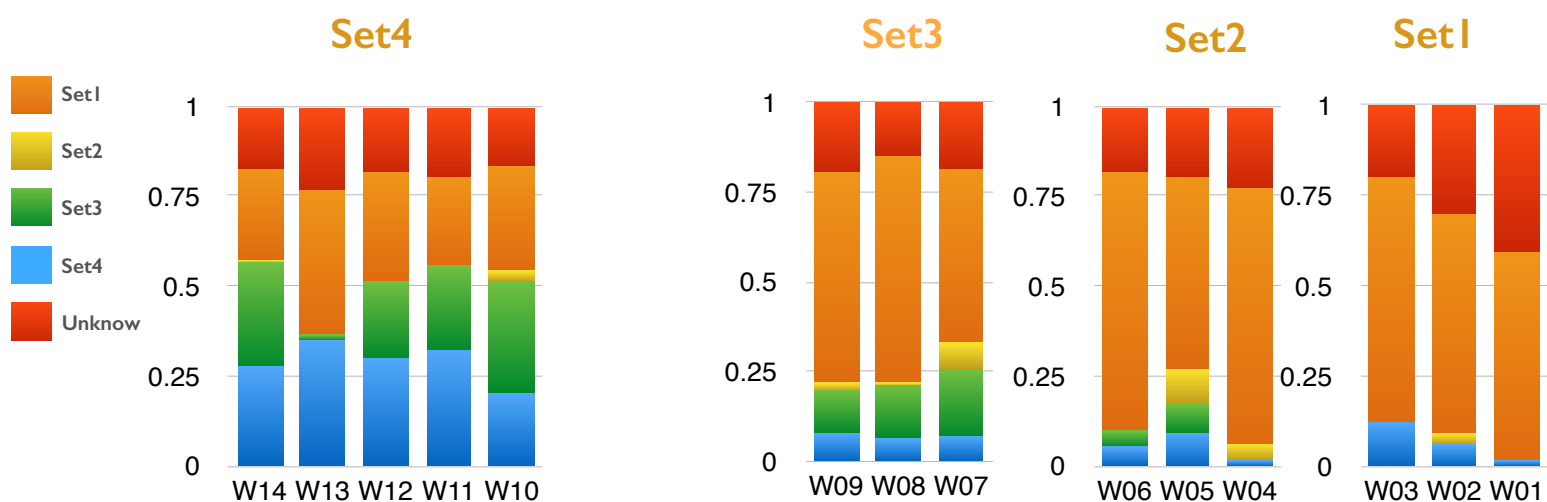

## PhyChem

Supplement: S3 Fig — This analysis demonstrates the proportion of the winter bacterioplankton that could be explained by samples from the summer. The Course results are in the upper panel, separated by the course categorization into upper, middle and lower. The bottom panel shows the results for the PhyChem analysis for each winter sample in the four different physicochemical sets (Set1–4). The results could be related to the middle panel illustrating the Sinos River and the sampling points. The colored bars for each winter sample represent the proportion of the bacterial structured populations that was present in the summer in that course of the river, based on the Course and PhyChem categorizations. (PDF) [file pone.0120608.s003.pdf]

S4 Fig.

# Alpha-diversity

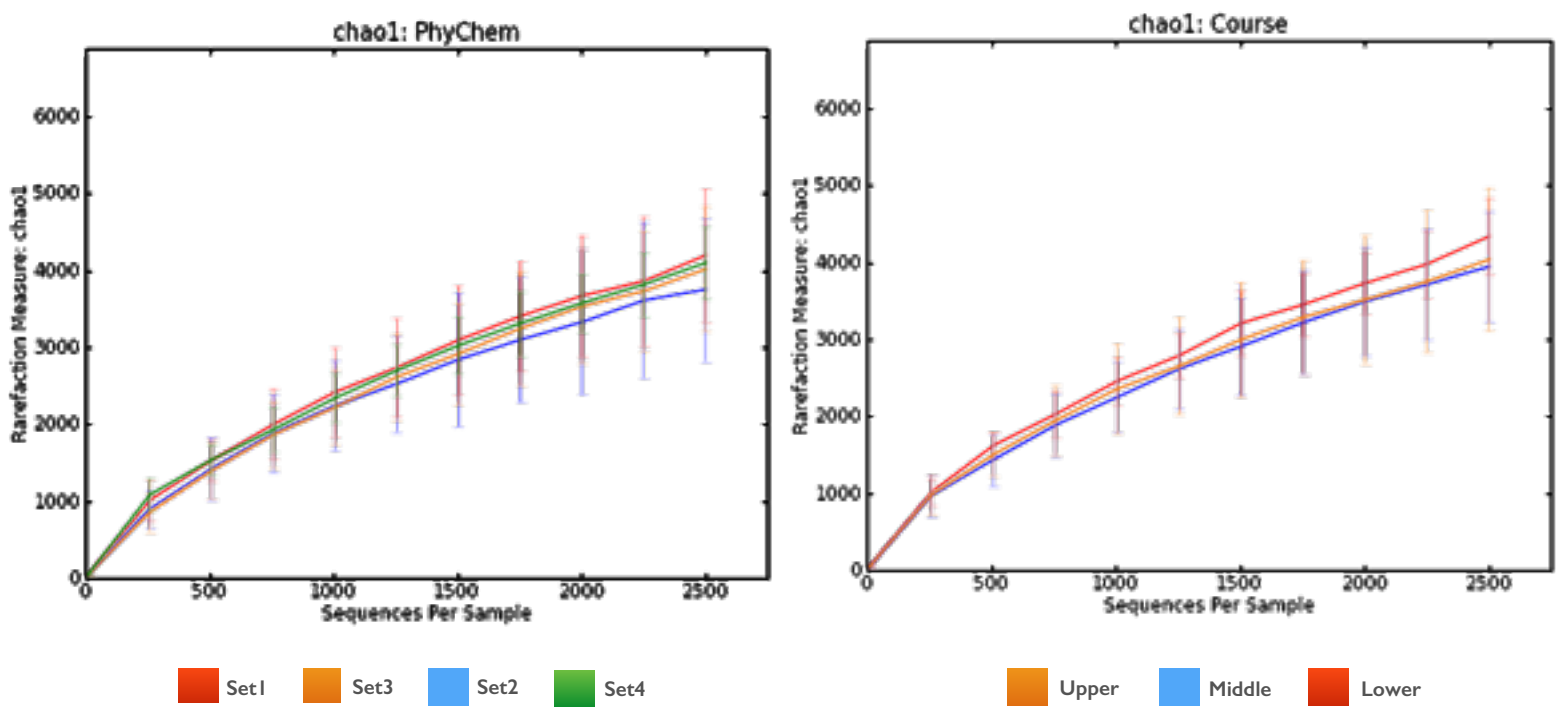

Supplement: S4 Fig — Plot of the alpha-diversity results for the summer and winter samples combined by the PhyChem and Course groups. (PDF) [file pone.0120608.s004.pdf]

# Alpha-diversity

Winter

Summer

PhyChem

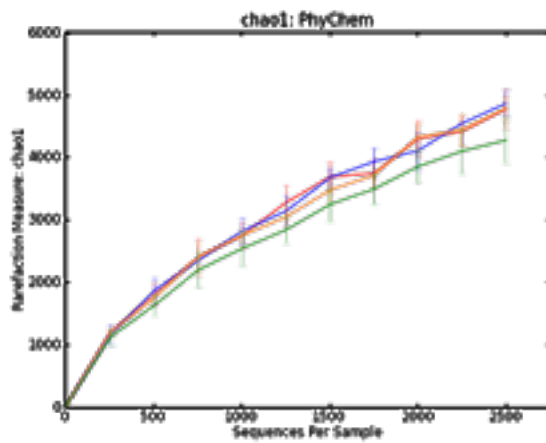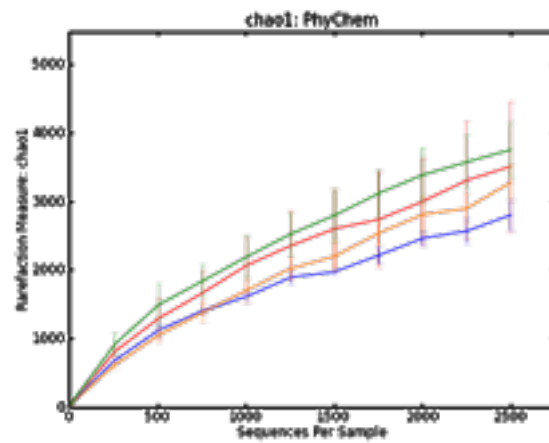

Set1

Set2

Set3

Set4

Cour

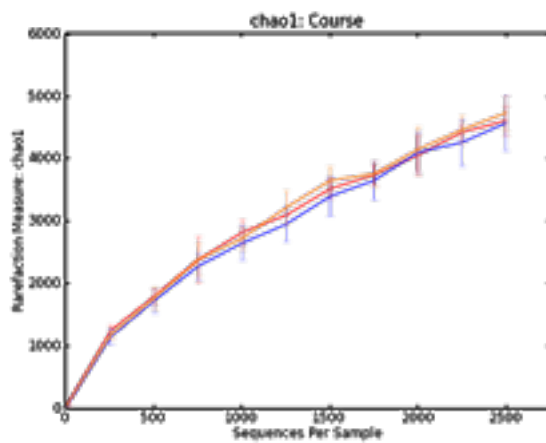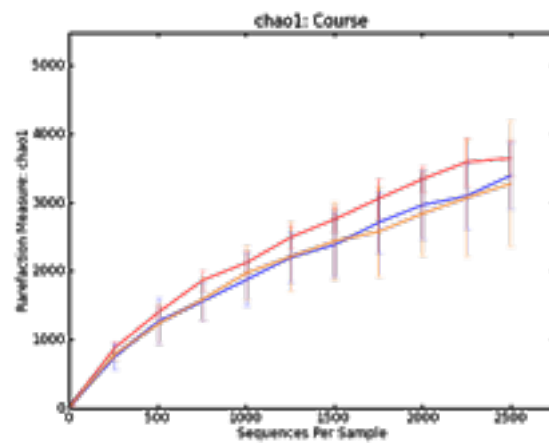

Upper

Middle

Lower

Supplement: S5 Fig — Plot representing the independent analysis of the samples for both seasons, summer and winter, by the Course and PhyChem categorizations. (PDF) [file pone.0120608.s005.pdf]

S6 Fig.

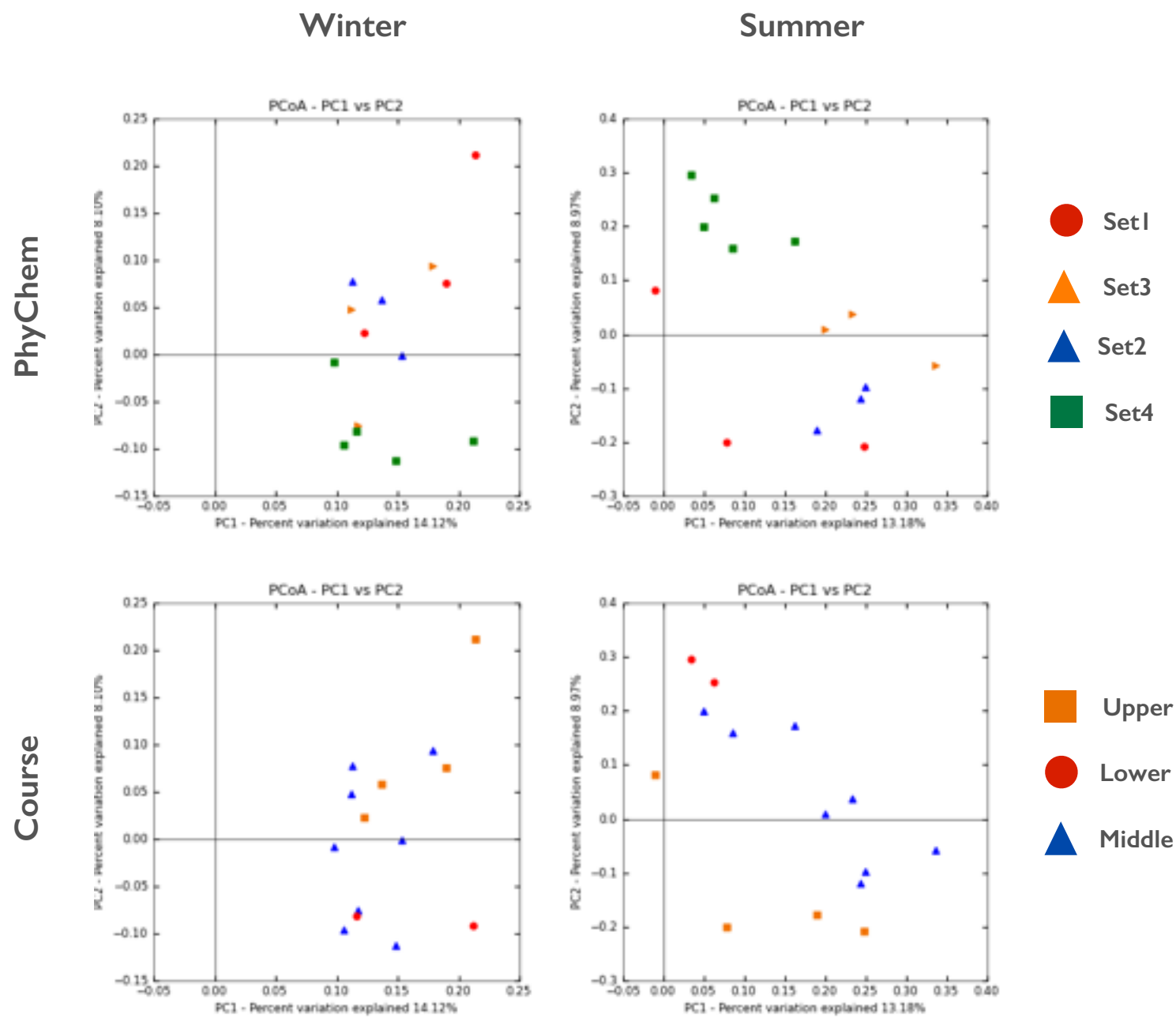

Supplement: S6 Fig — Principal Coordinate Analysis showing the correlations for the individual samples in both seasons, summer and winter, by the Sinos River PhyChem and Course categorizations. (PDF) [file pone.0120608.s006.pdf]

S7 Fig.

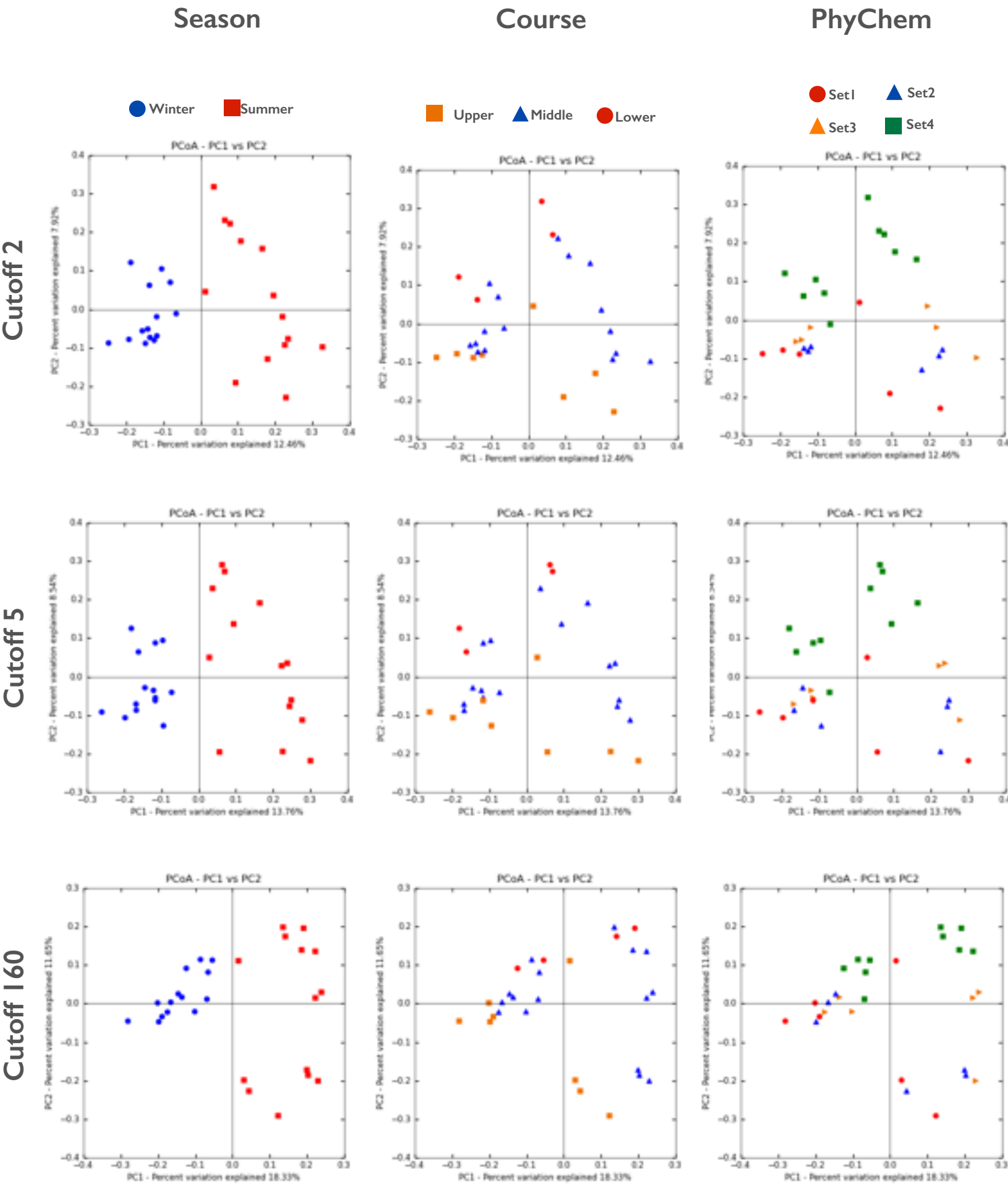

Supplement: S7 Fig — Principal Coordinate Analysis showing the correlations for the individual samples in both seasons, summer and winter, by the Sinos River PhyChem and Course categorizations applied to OTU cutoff of 2, 5 and 160 reads. (PDF) [file pone.0120608.s007.pdf]
